# Supplementary material for: Defining murine monocyte differentiation into colonic and ileal macrophages
Source: eLife. 2020 Jan 8;9:e49998. doi: 10.7554/eLife.49998 (PMC6952180; doi:10.7554/eLife.49998)
Supplement: Supplementary file 1. [file elife-49998-supp1.docx]

**Table Supplement 1**

List of RNAseq data sets prepared and used in this study

| **data set #** | **description** | **mice** | **number of repeats** | **main relevant figure(s)** | **comments** |
| --- | --- | --- | --- | --- | --- |
| 1 | Colonic macrophages | C57BL/ 6 | 4 | Figure 1 – figure supplement 1 |  |
| 2 | Ileum macrophages | C57BL/ 6 | 4 | Figure 1 – figure supplement 2 |  |
| 3 | Colon macrophages | C57BL/ 6 | 4 | Figure 1 – figure supplement 4 | data 3 , 4 are from same animals |
| 4 | Colonic ‘monocytes’ | C57BL/ 6 | 4 | Figure 1 – figure supplement 4 | data 3 , 4 are from same animals |
| 5 | BM monocytes (graft) | C57BL/ 6 CX3CR1-GFP (CD45.1) | 3 | Figure 1 |  |
| 6 | Colon, Graft-derived cells Day 4 | C57BL/ 6 CX3CR1-GFP (CD45.1) | 3 | Figure 1 | CD11c-DTR recipient |
| 7 | Colon, Graft-derived cells Day 8 | C57BL/ 6 CX3CR1-GFP (CD45.1) | 3 | Figure 1 | CD11c-DTR recipient |
| 8 | Colon, Graft-derived cells Day 12 | C57BL/ 6 CX3CR1-GFP (CD45.1) | 4 | Figure 1 | CD11c-DTR recipient |
| 9 | Ileum, Graft-derived cells Day 4 | C57BL/ 6 CX3CR1-GFP (CD45.1) | 4 | Figure 1 | CD11c-DTR recipient |
| 10 | Ileum, Graft-derived cells Day 8 | C57BL/ 6 CX3CR1-GFP (CD45.1) | 4 | Figure 1 | CD11c-DTR recipient |
| 11 | Ileum, Graft-derived cells Day 12 | C57BL/ 6 CX3CR1-GFP (CD45.1) | 4 | Figure 1 | CD11c-DTR recipient |
| 12 | Blood monocyte Ly6C+ | C57BL/ 6 | 3 | Figure 6 |  |
| 13 | Blood monocyte Ly6C- | C57BL/ 6 | 3 | Figure 6 |  |
| 14 | Colon, Graft-derived cells Day 14 | C57BL/ 6 CX3CR1-GFP (CD45.1) | 4 | Figure 2 – figure supplement 6 | CX3CR1-DTR recipient |
|  |  |  |  |  |  |
